# Choice of antipsychotic treatment by European psychiatry trainees: are decisions based on evidence?

**DOI:** 10.1186/1471-244X-12-27

**Published:** 2012-03-30

**Authors:** Sameer Jauhar, Sinan Guloksuz, Olivier Andlauer, Greg Lydall, João Gama Marques, Luis Mendonca, Iolanda Dumitrescu, Costin Roventa, Nele De Vriendt, Jeroen Van Zanten, Florian Riese, Izu Nwachukwu, Alexander Nawka, Raphael Psaras, Neil Masson, Rajeev Krishnadas, Umberto Volpe

**Affiliations:** 1 Chair, EFPT Research Group, Sackler Institute for Psychobiological Research, Institute of Neurological Sciences, Southern General Hospital, 2nd Floor 1345 Govan Rd, Glasgow G51 4TF, UK; 2 Department of Psychiatry and Psychology, Maastricht University Medical Centre, P.O. Box 616, 6200, MD Maastricht, The Netherlands; 3 EA 481 Laboratoire de Neurosciences, University of Franche-Comte, and Department of Clinical Psychiatry, University Hospital of Besancon, F-25030 Besancon, France; 4 Department of Molecular Psychiatry, Mental Health Sciences, University College London, London W1T 4JF, UK; 5 Centro Hospitalar Psiquiátrico de Lisboa, Psychiatry Educator at Faculdade de Medicina de Lisboa, Lisboa, Portugal; 6 Centro Hospit alar Psiquiatrico de Lisboa, Lisboa, Portugal; 7 Hospital Pr Dr Al Obregia, Bucharest, Romania; 8 Hospital Pr Dr Al Obregia, Bucharest, Romania; 9 Openbaar Psychiatrisch Zorgcentrum Rekem, Rekem, Belgium; 10 Department of Psychiatry, Neuroscience Campus Amsterdam, VU Medical Center, Amsterdam, the Netherlands; 11 Psychiatric University Hospital Zurich, Zurich, Switzerland; 12 St Vincent's University Hospital, Dublin 4, Ireland; 13 Department of Psychiatry, 1st Faculty of Medicine, Charles University in Prague, Ke Karlovu 11, 128 00 Praha 2, Prague, Czech Republic; 14 1st Psychiatric Department, Psychiatric Hospital of Attica, 374, Athinon avenue, 12462 Athens, Greece; 15 Wishaw Resource Centre, 48-54 Roberts Street, Wishaw, ML2 7JF, UK; 16 Sackler Institute for Psychobiological Research,, Institute of Neurological Sciences, Southern General Hospital, 2nd Floor 1345 Govan Rd, Glasgow G514TF, UK; 17 Department of Psychiatry, University of Naples - SUN Largo Madonna delle Grazie, 80138 Napoli, Italy

**Keywords:** Antipsychotics, Drug therapy, Psychiatry trainees, Evidence-based medicine, Decision-making, Efficacy, Psychotherapy, Psychosis, Treatment

## Abstract

**Background:**

Little is known about the factors influencing treatment choice in psychosis, the majority of this work being conducted with specialists (consultant) in psychiatry. We sought to examine trainees' choices of treatment for psychosis if they had to prescribe it for themselves, their patients, and factors influencing decision-making.

**Methods:**

Cross-sectional, semi-structured questionnaire-based study.

**Results:**

Of the 726 respondents (response rate = 66%), the majority chose second-generation antipsychotics (SGAs) if they had to prescribe it for themselves (n = 530, 93%) or for their patients (n = 546, 94%). The main factor influencing choice was perceived efficacy, 84.8% (n = 475) of trainees stating this was the most important factor for the patient, and 77.8% (n = 404) stating this was the most important factor for their own treatment. Trainees with knowledge of trials questioning use of SGAs (CATIE, CUtLASS, TEOSS) were more likely to choose second-generation antipsychotics than those without knowledge of these trials (χ^2 ^= 3.943; *p *= 0.047; O.R. = 2.11; 95% C.I. = 1.0-4.48). Regarding psychotherapy, cognitive behavioural therapy (CBT) was the most popular choice for self (33.1%; n = 240) and patient (30.9%; n = 224). Trainees were significantly more likely to prefer some form of psychotherapy for themselves rather than patients (χ^2 ^= 9.98; *p *< 0,002; O.R. = 1.54; 95% CIs = 1.18-2.0).

**Conclusions:**

Trainees are more likely to choose second-generation antipsychotic medication for patients and themselves. Despite being aware of evidence that suggests otherwise, they predominantly base these choices on perceived efficacy.

## Background

Historically, treatments in psychiatry have invariably been controversial, from the days of insulin coma and leucotomy to the use of psychotropic medication in the modern era. Recently the spotlight has fallen on use of antipsychotics (or more accurately, the *choice *of antipsychotic medication) for the treatment of psychosis.

The inception of Chlorpromazine, in 1950, signified a paradigm shift in the management (and our understanding) of schizophrenia. Although several other antipsychotics were developed over the following years, it took till the 1980s for clear evidence of benefit for one specific antipsychotic (Clozapine) to emerge [1]. Subsequently, another class of antipsychotics, the (so-called) "atypical" antipsychotics (or second-generation antipsychotics (SGA)) were developed. At the time of their introduction these drugs were promoted as having superior efficacy and better side-effect profiles to their older counterparts [2]. This came at a price: these drugs were initially priced much higher than their older counterparts, though, guidelines continued to recommend their use [3].

However, following the publication of at least two recent large effectiveness trials [4,5], these assumptions have been significantly challenged. Moreover, the effectiveness of SGAs (with the exception of Clozapine) has been shown to be roughly equivalent to their first-generation counterparts, with differing side-effect profiles, with one recent trial being discontinued on account of metabolic side-effects observed with one of the SGAs [6].

Despite this evidence, it is difficult to tell how any of the recent notions of efficacy and tolerability of SGAs have impacted on clinical practice, and if this has influenced actual clinical decision-making. In contrast to the burgeoning literature on antipsychotics, relatively few studies have examined decision-making in the context of psychosis. Methods used for this include semi-structured interviews with psychiatrists, and preference for psychiatrists' own treatment [7-9]. The majority of this work has been conducted locally (in one case a national survey), predominantly with psychiatry consultants/specialists. To date, there has been little work examining trainee psychiatrists' views on treatment, and no examination of their views on psychotherapy. Moreover, populations that have been looked at have only included those from the United Kingdom and Germany [8-10]. Thus, further work is warranted.

The aim of this study was to ascertain choices of psychiatric trainees from various European countries regarding antipsychotic treatments, the factors influencing their choices, and whether treatment choices were altered when trainees were asked which treatment they would choose for their *own *care.

## Methods

### Study participants

Participants were given a guarantee that every attempt would be made to ensure that responses to the questionnaire would be confidential. In view of these conditions, returning a pseudo-anonymised questionnaire was considered to be indicative of informed consent. These considerations are in keeping with the ethical principles set out in the Declaration of Helsinki [11].

### Inclusion criteria

To ensure adequate reliability, a minimum sample of 50 trainees from each country was agreed on. Each country participant was asked to sample a group of trainees that included trainees from a similar institution or area, and this represented an opportunistic sample. A minimum response rate for inclusion into the study was set (50%).

### Survey

Based on guidelines for surveys [12] and prior work [9,10] that utilised semi-structured questionnaires asking about choice for oneself, patients and factors influencing choice, an *ad hoc *semi-structured survey was developed. This survey was piloted with psychiatric trainees attending the EFPT (European Federation of Psychiatric Trainees) Annual Forum. The survey was distributed in English, via a web-link and via paper copy, for six months, between October 2008 and March 2009. During this period, country representatives were regularly updated on response rates to the web survey, and were permitted to contact participants. The full survey is given in Additional file 1: Appendix 1, and included

i) Demographic details (gender, country, adult or child and adolescent trainee, years of training).

ii) Antipsychotic choice for patients presenting with a psychotic episode lasting longer than one month (typical, atypical).

iii) Generic drug name for antipsychotic chosen.

iv) Factors influencing choice (cost, efficacy, side effect profile, other).

v) Whether any recent trials had influenced decision-making (given choice of CATIE, CUtLASS, TEOSS or other). These trials were chosen on account of their topical nature, large numbers and perceived influence.

vi) Whether adjunctive psychotherapy would be considered (and reasons for doing so).

vii) Trainees' choice of antipsychotic if *they *developed a psychotic episode lasting one month or longer (class, generic drug) and factors influencing choice, and adjunctive psychotherapy.

Through the web forum, agreement was reached on operational definitions (and understanding) of the terms "antipsychotic", "typical antipsychotic", "atypical antipsychotic" and "psychotic episode".

### Analysis

Output from the web-survey tool was analysed using Predictive Analytics Software Statistics Programme (PASW; version 18.0). Given the exploratory nature of our analysis, chi-square tests, or Fisher's exact tests when relevant, were used to compare categorical data. Statistical significance was set at *p *< 0.05. The qualitative responses on choice of psychological treatments were analysed using content analysis, with comparisons being made between the reasons for the treatment chosen, according to set criteria. This was performed separately by SG, and SJ and NM, and the results were compared and revised to increase reliability.

## Results

Of the 18 countries originally participating in the survey, only 12 met inclusion criteria listed above. Countries that participated but were not included were Croatia, Germany, Italy, Russia, Spain and Sweden. These countries were excluded on account of being unable to collect samples meeting the inclusion criteria. Reasons for this included "research fatigue" (a number of other surveys were being conducted at the current time).

The included countries and response rates are given in Table 1.

**Table 1:** Countries meeting inclusion criteria and response rates

Country	Number of participants/Total number sampled	Response rate (%)
Belgium	52/104	50%

Czech Republic	60/92	65%

England	53/98	54%

France	60/100	60%

Greece	75/100	75%

Holland	54/75	72%

Ireland	51/72	71%

Portugal	52/94	40-55%^1^

Romania	74/99	75%

Scotland	54/75	72%

Switzerland	83/124	67%

Turkey	58/70	83%

Total	726/1103	66%

(Countries that participated but were not included were Croatia, Germany, Italy, Russia, Spain and Sweden). ^1^Estimated response rate, based on variable reliability of email addresses

### Demographic characteristics

Of the 726 trainees, 84.4% (n = 613) were adult psychiatry trainees and 15% (n = 109) were child and adolescent psychiatry trainees. 54.8% (n = 398) of the sample were females. The mean duration of completed training was 2.97 (s.d. = 1.57) years. More than half of the trainees were in the first half of their training (n = 438; 60%).

### Oral antipsychotic choice

**Figure 1:**
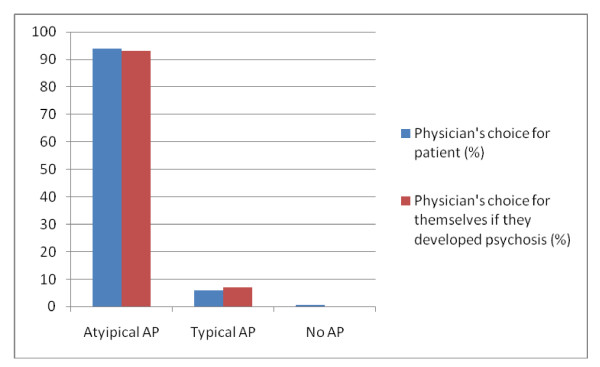
**Type of antipsychotic chosen by trainees, for patients or for trainees themselves**.

Antipsychotic choice for patients and trainees themselves, grouped by class and generic name, are presented in Table 2. Given recent evidence, underlining that the "atypical" and "typical" antipsychotics are heterogeneous entities [13], and that some atypical antipsychotics may have more efficacy than others, we examined choices of specific antipsychotics. Regarding the class of antipsychotic (atypical, typical or no antipsychotic), there were no differences between trainees' treatment choices for their patients or for themselves (Figure 1).

**Table 2:** Antipsychotics chosen by trainee in given scenario and for own care

Antipsychotic chosen	Physician's choice for patient	Physician's choice if they developed psychosis
Atypical	546 (94%)	530 (93%)

Typical	35 (6%)	40 (7%)

No antipsychotic	4 (0.7%)	0

Olanzapine	167 (210)	39.9% 100 (120), 26.2%

Risperidone	154 (202)	36.8% 123 (141), 32.3%

Haloperidol	28 (33)	6.7% 21 (23), 5.5%

Aripiprazole	25 (33)	6% 65 (73) 17%

Amisulpiride	17 (21)	4.1% 19 (24) 5%

Quetiapine	22 (36)	5.3% 25 (37) 6.6%

Clozapine	6 (8)	1.4% 22 (24) 5.8%

No response given regarding generic drug	202	304

Electroconvulsive therapy (ECT)	0	1 0.3%

Ziprasidone	0	4 1%

Sertindole	0	1 0.3%

Percentages given are of those who responded to the question. Some respondents chose one or more antipsychotics concurrently; numbers in brackets include these additions. The table illustrates that the "atypical" class of antipsychotics were the most popular choice for patients and trainees themselves

**Figure 2:**
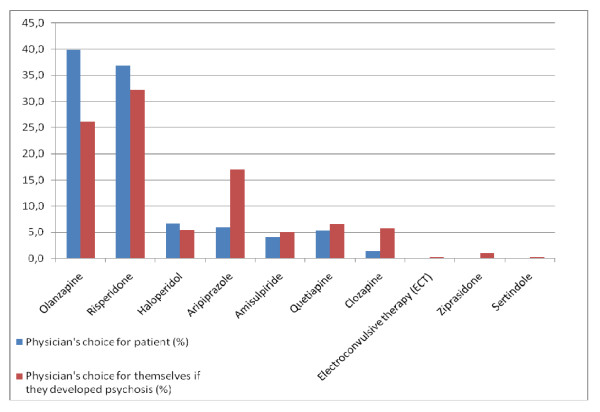
**Percentages of drug chosen by trainees, for patients or for trainees themselves**.

Olanzapine was the most popular choice for the patient in the scenario, and Risperidone the most popular choice for trainees themselves (Figure 2). Trainees were more likely to prescribe olanzapine for patients (χ^2 ^= 16.83, *p *< 0.0001, O.R. = 1.9, 95% C.I. = 1.38-2.53), and aripiprazole for themselves (χ^2 ^= 24.48, *p *< 0.001, O.R. = 3.2, 95% C.I. = 2.0-5.2). Only one typical antipsychotic drug (haloperidol) was chosen. Forty-five (6%) trainees did not choose a generic antipsychotic, stating that the decision would depend on characteristics of the patient and their symptom profile (e.g., need for sedation and side-effects experienced). No difference was seen between males and females for antipsychotic choice for patient or self.

### Differences between countries

Differences between countries and antipsychotic choice were examined, using chi-square tests. Trainees from Holland were more likely to prescribe typical antipsychotics for patients than those from other countries, (χ^2 ^= 16.18; *p *= 0.01; O.R. = 4.76; 95% C.I. = 2.08-10.86). Dutch trainees were also more likely to choose typical antipsychotics for their own treatment, compared to trainees from other countries, (χ^2 ^= 51.6; *p *< 0.001; O.R. = 10.9; 95% C.I. = 5-23.8). A similar finding was found with trainees from Turkey, in regard to treatment for the patient in the given scenario, (χ^2 ^= 32.35; *p *< 0.001; O.R. = 6.90; 95% C.I. = 3.26-14.7).

### Antipsychotic class

528 trainees filled in both questions "antipsychotics for self" and "antipsychotics for patients". Trainees were no more likely to prescribe an atypical for themselves (93.6%) than they would for patients (94.5%) (χ^2 ^= 0.42; *p *= 0.5, OR = 0.84 (95% C.I. = 0.5-1.4). As detailed above there were multiple entries for specific antipsychotics, and therefore it was not possible to include these in the calculation of odds ratios, as those who selected two antipsychotics did not consider one to be superior to the other.

### Factors influencing choice of antipsychotic

Factors influencing choice mapped onto the three main domains given in the questionnaire: cost, efficacy and side-effect profile. Less than 5% of trainees gave other reasons. Results are summarised in Table 3. Efficacy was the most important factor influencing choice for treating patients as well as for oneself, followed by side effect profile and then cost. When considering their own treatment (as opposed to a patient), trainees were significantly more inclined to think of side-effect profile (χ^2 ^= 15.49; *p *< 0.0001; O.R. = 1.64; 95% C.I. = 1.28-2.10), and less inclined to think of efficacy (χ^2 ^= 8.69; *p *= 0.03; O.R. = 0.63; 95% C.I. = 0.46-0.86). Factors influencing specific antipsychotic choice are summarized in Table 4. Trainees favoured efficacy over side-effect profile for all drugs, except Aripiprazole for patients and themselves, and Quetiapine for themselves. For these two drugs, side-effect profile was the most important criterion.

**Table 3:** Factors influencing choice of antipsychotic for patient/self

	Efficacy		Side-effect profile		Cost	
	
	Patient	Self	Patient	Self	Patient	Self
Most important	475 (84.8%)	404 (77.8%)	191 (34.5%)	237 (46.4%)	17 (3.2%)	13 (2.7%)

2^nd ^most important	84 (15%)	115 (22.2%)	326 (59%)	258 (50.1%)	91 (17.2%)	69 (14.2%)

Least important	1 (0.2%)	0	36 (6.5%)	16 (3.1%)	422 (79.6%)	405 (83.2%)

Respondents could choose more than one factor at each stage of importance; percentages reported are for those responding to the question

**Table 4:** Factors influencing the choice of a specific antipsychotic for patient/self

	Olanzapine		Risperidone		Haloperidol		Quetiapine		Aripiprazole		Amisulpiride		Clozapine	
	
	P	S	P	S	P	S	P	S	P	S	P	S	P	S
**Efficacy** **most** **important**	155 (92.8%)	93 (93%)	129 (83.8%)	118 (76.1%)	23 (82.1%)	15 (78.9%)	16 (72.7%)	14 (56%)	10 (40%)	32 (49.2%)	16 (94.1%)	16 (84.2%)	6 (100%)	20 (90.9%)

**Side effect** **profile most** **important**	38 (22.8%)	25 (25%)	39 (25.3%)	70 (45.2%)	6 (21.4%)	6 (31.6%)	13 (59.1%)	19 (76%)	16 (64%)	44 (67.7%)	4 (23.5%)	7 (36.8%)	0	3 (13.6%)

P refers to patient; S refers to self

(Note: some trainees chose more than one factor at a time)

### Prior knowledge of effectiveness trials

The percentage of trainees who reported that prior knowledge of effectiveness trials (CATIE, CUtLASS and TEOSS) had influenced their antipsychotic choice was 33.8% (n = 246), 8.1% (n = 59) and 1.5% (n = 11), respectively. Trainees with prior knowledge of at least one effectiveness trial chose atypical antipsychotics for the patient in the scenario more than trainees with no prior knowledge of any of these effectiveness trials (96.1% versus 92.3%; χ^2 ^= 3.943; *p *= 0.047; O.R. = 2.11; 95% C.I. = 1.0-4.48).

### Influence of trainee seniority and type of trainee

No differences in class of antipsychotic chosen for patients were seen between trainees in the first and second halves of training (92.3% versus 95.3%; χ^2 ^= 2.12; *p *= 0.15; O.R. = 1.70; 95% C.I. = 0.83-3.3.40), and no statistically significant difference was observed between child and adolescent psychiatry trainees and adult trainees (97.4% versus 93.4%; Fisher exact *p *= 0.13).

### Choice of adjunctive psychotherapy

Choices of psychotherapy for both the patient in the scenario and for oneself are given in Table 5. Cognitive behavioural therapy (CBT) was the most popular choice for self and patient. Trainees were significantly more likely to prefer some form of psychotherapy for themselves rather than patients (χ^2 ^= 9.98; *p *< 0,002; O.R. = 1.54; 95% C.I. = 1.18-2.0). Psychodynamic psychotherapy was chosen more by trainees for themselves than for the patient in the scenario (χ^2 ^= 15.53; *p *< 0.001; O.R. = 2.63; 95% C.I. = 1.6-4.3).

**Table 5:** Choice of psychotherapy for patient/self

	Treatment for patient	Treatment for self
**None**	187 (25.8%)	133 (18.7%)

**Any kind of psychotherapy**	331	362**

**Cognitive Behavioural Therapy (CBT)**	240 (33.1%)	224 (30.9%)

**Interpersonal Therapy**	50 (6.9%)	56 (7.7%)

**Mindfulness-based Therapy**	17 (2.3%)	26 (3.5%

**Psychodynamic Psychotherapy**	24 (3.3%)	56 (7.7%)***

**Not responded**	208 (28.7%)	231 (31.8%)

**: *p *< 0.01; ***: *p *< 0.001

### Qualitative analysis of psychotherapy

289 trainees gave qualitative responses. There was no difference in the qualitative responses between psychotherapy treatments chosen by trainees for themselves or for their patients. The factors influencing a trainee not to use psychotherapy included a lack of evidence base/guidelines (n = 6), clinical experience (n = 9), lack of access to the treatment (n = 2), the opinions and practice of senior psychiatrists (n = 2) and the fact that the patient may be too unwell (n = 3). Many trainees described the usefulness of using unstructured forms of therapy such as supportive psychotherapy and of waiting until the psychotic symptoms had settled before considering therapy. Factors influencing choice of CBT included evidence base/guidelines (n = 63), availability (n = 2), low cost (n = 3), structured and less intensive approach (n = 2). Reasons for interpersonal therapy included allowing patients to cope (n = 3). Their clinical experience of using mindfulness-based treatment (n = 3) was used as a reason for its use.

Those favouring psychodynamic psychotherapy did so mostly because that was the treatment they were trained in (n = 6), with little mention of its evidence base (n = 2). As some trainees were already receiving psychodynamic psychotherapy they stated they would want this to continue if they developed a psychotic episode (n = 11).

## Discussion

### Pharmacotherapy

Our results suggest trainees prefer to prescribe, and receive SGAs, based on assumptions of improved efficacy and side-effect profile. These assumptions have been challenged by recent evidence [13]. What is striking is that, despite knowledge of recent evidence (trials that suggest small difference between classes), trainees would *not *change their prescribing practice. Although they would broadly choose the same class of antipsychotic for patients and themselves, some would wish to be treated with differing antipsychotics to their patients, basing this decision on side-effect profile. There was a general trend for more emphasis on side-effect profiles when the treatment was prescribed for trainees themselves.

The results regarding antipsychotics are similar to those of other studies, conducted in England, Germany and Scotland [8-10]. In all the previous studies "atypical" antipsychotics were preferred, with olanzapine and risperidone the most popular choices.

Factors influencing choice are generally similar to those reported elsewhere, with efficacy and side-effect profile/tolerability viewed as most important [8,9], a naturalistic study finding that physicians were more likely to base antipsychotic choice in clinical practice on side-effects [14]. This was reflected in our sample, when trainees chose their medication they would prefer to receive (significantly less choosing olanzapine and significantly more choosing aripiprazole), a possible reason for this being the difference in side-effects between these compounds, though this was not examined in further detail.

Decision-making was also examined in face to face interviews with German psychiatrists, the only distinguishing factor amongst participants being age, with older doctors being more likely to prescribe typical antipsychotics [7]. This was not found in our study, though we would suggest trainees with more experience have no significant exposure to the older compounds, unlike those in the German study.

### Psychotherapy

Findings regarding psychotherapy are that trainees chose to receive therapy for themselves more often than for patients, and that this was based on personal experience. CBT was the most popular choice, and evidence base was cited as the reason for this. A number of trainees who had received psychodynamic therapy for themselves wanted to receive this if they developed psychosis. Though did not choose this for the patient in the given scenario.

### "Evidence-based medicine"

The finding in regard to knowledge of effectiveness trials is counter-intuitive, and may reflect concerns trainees may have had with the trials themselves, and their interpretation of the results, owing to methodological issues, that have been highlighted elsewhere [15]. It may also reflect wider concerns, regarding the philosophical underpinnings of EBM itself, which have been eloquently argued elsewhere [16]. Briefly, this consists of theoretical constructs (EBM) versus clinical observation. This probably has relevance to our sample, as a number of trainees stated that both clinical experience and evidence had affected their choice of psychotherapy.

To our knowledge this is the only study that has examined choice of psychotherapy in psychosis, none of the above studies examining this question. This area is worth examining, especially in the context of recent literature that questions efficacy of CBT in psychosis, querying if any benefit is seen in well-conducted studies [17], and recent guidelines that advocate for its use.

The finding that physicians would have differing preferences for their own care has been examined in detail in a recent German study of 515 psychiatrists, who were randomised to various groups [18]. In that study, when given a scenario involving a patient with a relapse of psychosis in schizophrenia, physicians chose watchful waiting and oral antipsychotic medication, as opposed to depot antipsychotic medication when asked about treatment for a patient, or themselves.

Weaknesses of this study pertain to its design (cross sectional, arbitrary survey with an opportunistic sample), which lacks the thoroughness and validity of a face-to-face interview. The results from all the countries were pooled, but we have to keep in mind that the training systems, health systems, and the availability of the drugs, are different between the different countries. Although efforts were made to recruit an adequate sample that was felt to be representative, the fact that only a small proportion of the total trainees in Europe were surveyed tempers the generalisability of these results. Furthermore, the validity of a sample of 50 in a country like Portugal would be greater than those for a country like England, which has significantly more trainees.

We would point out, however, that this is, to our knowledge, the largest study conducted examining choice, and the only one involving exclusively psychiatric trainees. This is relevant since most psychiatry trainees have had more exposure to the second-generation antipsychotics [19], and have less clinical experience to base their decisions on. Moreover, psychiatric trainees are tomorrow's psychiatrists, and therefore these results give us an overview of what future European prescription patterns might be. We were also in a favorable position to ask about changes in the recent evidence-base, owing to the close proximity of the questionnaire to the findings from effectiveness trials like CATIE.

## Conclusions

European psychiatry trainees appear to base treatment decisions on factors other than purely evidence-base, and would choose similar treatments for psychosis (atypical antipsychotics) for themselves and their patients. Differences appear to exist in the individual compounds they would choose to receive for themselves, possibly reflecting concerns about side-effects, such as weight gain. Future work would focus on what influences perceptions of efficacy and side effect profile, and what role the pharmaceutical industry and opinion leaders may play in these assumptions.

## Competing interests

SJ, SG, GL, JM, LM, ID, CR, NDV, NI, JVZ, AN, RP, NM, RK, UV declare no conflict of interest. FR has received a speaker's fee from Lundbeck (Switzerland). In the past five years, OA has received reimbursements, fees or funding from: Eli Lilly, Lundbeck, BMS/Ostuka, Janssen-Cilag.

## Authors' contributions

SJ, SG, OA, GL, JGM, LM, ID, CR, AW, IN, FR, JVZ, NDV and RP carried out data collection, and contributed to the writing of the manuscript. SJ, SG, NM and RK carried out data analysis and the writing of the manuscript. UV carried out interpretation of the data and writing of the manuscript. All authors read and approved the final manuscript.

## Authors' information

The EFPT Research group is a working group, under the umbrella of the European Federation of Psychiatric Trainees, a trainee-led, non-governmental organisation, dedicated to improving training and education of psychiatric trainees across Europe. The Research group was incepted to provide meaningful research opportunities for psychiatry trainees from different European countries, and to foster collaboration on a European basis.

## Pre-publication history

The pre-publication history for this paper can be accessed here:

http://www.biomedcentral.com/1471-244X/12/27/prepub

## Supplementary Material

Additional file 1 **Appendix 1**. Click here for file
