# Supplementary material for: Choice of antipsychotic treatment by European psychiatry trainees: are decisions based on evidence?
Source: BMC Psychiatry. 2012 Mar 30;12:27. doi: 10.1186/1471-244X-12-27 (PMC3337226; doi:10.1186/1471-244X-12-27)
Supplement: Additional file 1 — Appendix 1. [file 1471-244X-12-27-S1.DOC]

**Appendix 1**

1. You have a patient with a psychotic episode lasting more than one month. Which one class of antipsychotic would you prescribe?

Typical/atypical (including second generation)/none

2. Please specify the generic drug name you would choose (or explain why you chose none)

3. What influenced your decision?

|  | Most important | 2^nd^ most important | Least important |
| --- | --- | --- | --- |
| Cost |  |  |  |
| Efficacy (power to produce a desired effect) |  |  |  |
| Side-effect profile |  |  |  |

Other (please specify)

4. Did any of the evidence below influence your decision?

CATIE/CUtLASS/TEOSS

Other (please specify)

5. Which of the following forms of psychotherapy (if any) would you recommend in the above patient?

CBT

Interpersonal therapy

Mindfulness based therapy

Psychodynamic psychotherapy

None

Other (please specify)

(Next page)

6. If you had a psychotic episode lasting more than one month, which one class of antipsychotic would you prescribe?

Typical/atypical (including second generation)/none

7. Please specify the generic drug name you would choose (or explain why you chose none)

8. What influenced your decision?

|  | Most important | 2^nd^ most important | Least important |
| --- | --- | --- | --- |
| Cost |  |  |  |
| Efficacy (power to produce a desired effect) |  |  |  |
| Side-effect profile |  |  |  |

Other (please specify)

9. Did any of the evidence below influence your decision?

CATIE/CUtLASS/TEOSS

Other (please specify)

10. Which of the following forms of psychotherapy (if any) would you recommend in the above patient?

CBT

Interpersonal therapy

Mindfulness based therapy

Psychodynamic psychotherapy

None

Other (please specify)
